# The rs1800469 T/T and rs1800470 C/C genotypes of the TGFB1 gene confer protection against diabetic retinopathy in a Southern Brazilian population

**DOI:** 10.1590/1678-4685-GMB-2022-0247

**Published:** 2023-07-07

**Authors:** Aline Rodrigues Costa, Cristine Dieter, Luís Henrique Canani, Taís Silveira Assmann, Daisy Crispim

**Affiliations:** 1 Hospital de Clínicas de Porto Alegre, Serviço de Endocrinologia e Metabologia, Porto Alegre, RS, Brazil.; 2 Universidade Federal do Rio Grande do Sul, Faculdade de Medicina, Departamento de Medicina Interna, Programa de Pós-Graduação em Ciências Médicas: Endocrinologia, Porto Alegre, RS, Brazil.

**Keywords:** Diabetic retinopathy, polymorphisms, rs1800470, rs1800469, TGFB1

## Abstract

The transforming growth factor beta 1 (TGFB1) is a pro-inflammatory cytokine that plays a key role in the mechanisms of angiogenesis and breakdown of the blood-retina barrier, which are implicated in the pathogenesis of diabetic retinopathy (DR). Polymorphisms in the *TGFB1* gene have been associated with DR; however, results are still contradictory. Therefore, the aim of this study was to investigate the potential association between two *TGFB1* polymorphisms and DR. This study included 992 patients with diabetes mellitus (DM): 546 patients with DR (cases) and 446 patients without DR and with ≥10 years of DM (controls). The *TGFB1* rs1800469 and rs1800470 polymorphisms were genotyped by real-time PCR. Frequency of rs1800469 T/T genotype was higher in controls compared to DR cases (18.3% *vs*. 12.7%, P= 0.022). This genotype remained associated with protection for DR, adjusting for covariables (OR= 0.604; 95% CI 0.395 - 0.923; P= 0.020, recessive model). The rs1800470 C/C genotype was observed in 25.4% of the controls and 18.0% of the cases (P= 0.015); thus, being associated with protection against DR under the recessive model (OR= 0.589; 95% CI 0.405 - 0.857; P= 0.006), adjusting for covariables. In conclusion, the *TGFB1* rs1800469 and rs1800470 polymorphisms are associated with protection against DR in DM patients from Southern Brazil.

## Introduction

Diabetic retinopathy (DR) is a common chronic microvascular complication of diabetes mellitus (DM) and represents the primary cause of visual impairment and loss in working-aged adults (Cheung et al., 2010; Solomon et al., 2017; Kusuhara et al., 2018). DR affects approximately 35% of DM patients, being more frequent in type 1 DM (T1DM) than in type 2 DM (T2DM) patients (Yau et al., 2012). Its prevalence increases with DM duration, with ≅ 86% of T1DM and 52% of T2DM patients showing some degree of DR after 20 years of DM duration (Yau *et al.,* 2012). Although the risk of DR increases with poor glycemic control, long-term DM, arterial hypertension (AH), dyslipidemia, and body mass index (BMI), available evidence has suggested its development is also influenced by genetic factors (Cho and Sobrin, 2014; Priščáková et al., 2016; Han et al., 2019). In this context, chronic hyperglycemia and other risk factors initiate a cascade of biochemical and physiological alterations that can culminate in microvascular damage and subsequent retinal dysfunction. These changes are linked to retinal ischemia, abnormal angiogenesis, and increased vascular permeability due to breakdown of the blood-retina barrier (Cheung *et al.,* 2010; Kusuhara *et al.,* 2018). 

The transforming growth factor beta 1 (TGFB1) is a pro-fibrotic and pro-inflammatory cytokine that modulates cell proliferation, differentiation, apoptosis, adhesion, and migration of several cell types, and induces the production of extracellular matrix (ECM) proteins (Loeffler and Wolf, 2014). Given its critical roles in angiogenesis, endothelial proliferation, ECM deposition, and breakdown of the blood-retina barrier, *TGFB1* represents a candidate gene for susceptibility to DR as well as other chronic diabetic complications, including diabetic kidney disease (DKD) (Khan and Chakrabarti, 2003; Jia et al., 2011; Liu et al., 2014). Accordingly, several studies have associated single nucleotide polymorphisms (SNPs) in the *TGFB1* gene with susceptibility for DR and/or DKD (Beránek et al., 2002; Buraczynska et al., 2007; Jia *et al.,* 2011; Bazzaz et al., 2014; Liu *et al.,* 2014; Hampton et al., 2015; Zhou et al., 2018; Zhou *et al.,* 2019). 

The T allele of rs1800470 (c.+29 T>C, Leu10Pro) SNP in the *TGFB1* gene was initially associated with risk for proliferative DR (PDR) in patients with T2DM from the Czech population (Beránek et al., 2002). Conversely, another study reported that the C allele conferred risk for DR in patients with T2DM from Poland (Buraczynska et al., 2007). In 2014, Liu et al. (Liu et al., 2014) published a meta-analysis including 3 studies that investigated the association between the rs1800469 (c.-1347 C>T) SNP and DR; however, no significant association was found. Beránek *et al.* (Beránek *et al.,* 2002) reported that a haplotype constituted by both rs1800470 T and rs1800469 C alleles conferred increased risk for PDR. Due to the contradictory results, additional studies are needed to clarify whether these SNPs are associated with DR. 

Therefore, as part of the ongoing effort to examine the hypothesis that *TGFB1* SNPs are associated with DR, this study aims to investigate the association of rs1800469 (c.-1347 C>T) and rs1800470 (c.+29 T>C) SNPs in the *TGFB1* gene with DR in both T1DM and T2DM from a Southern Brazilian population.

## Material and Methods

### DM patients, phenotype measurements, and laboratory analyses

This case-control study was designed following STROBE and STREGA guidelines for reporting genetic association studies (von Elm et al., 2008; Little et al., 2009). The study population consisted of 992 DM patients, including 546 cases with DR and 446 controls without this complication and with a known DM duration of at least 10 years. Of note, of the total sample with DM, 727 (73.3%) patients had T2DM and 156 patients had T1DM (26.7%). All included patients were recruited from the outpatient clinic at the Hospital de Clínicas de Porto Alegre (Rio Grande do Sul, Brazil) between January 2005 and December 2013 (Crispim et al., 2010; Massignam et al., 2020). The research protocol was approved by the Ethics Committee in Research from Hospital de Clínicas de Porto Alegre, and all subjects provided assent and written informed consent prior to the inclusion in the study.

Patients were diagnosed as having DM according to American Diabetes Association guidelines (American Diabetes Association, 2020). Assessment of DR was performed by an experienced ophthalmologist using fundoscopy through dilated pupils. DR was classified as ‘absent DR’ (no fundus abnormalities), non-proliferative DR (NPDR, presence of microaneurysms, intraretinal hemorrhages, and hard exudates) or proliferative DR (PDR, newly formed blood vessels and/or growth of fibrous tissue into the vitreous cavity). DR classification was done considering the most severely affected eye, according to the Global Diabetic Retinopathy Group scale (Wilkinson et al.,, 2003). 

A standard questionnaire was used to collect information about age, age at DM diagnosis, type and DM duration, and drug treatment. Moreover, all patients underwent complete physical and laboratory evaluations, as previously reported by our group (Crispim et al., 2010; Bouças et al., 2013; Massignam et al., 2020). Ethnicity was defined based on self-classification, and patients were categorized in white and non-white subjects (Crispim *et al.,* 2010). Serum and plasma samples were taken after 12 h of fasting for laboratory analyses. Glucose levels were determined using the glucose oxidase method. Glycated hemoglobin (HbA1c) levels were measured by different methods and the results were traceable to the Diabetes Control and Complications Trial (DCCT) method by off-line calibration or using a conversion formulae (Camargo et al., 1998). Creatinine was measured by the Jaffé reaction; total plasma cholesterol, HDL cholesterol and triglycerides by enzymatic methods, and urinary albumin excretion (UAE) by immunoturbidimetry (Sera-Pak immuno microalbuminuria, Bayer, Tarrytown, NY, USA) (Zelmanovitz et al., 1997). The estimated glomerular filtration rate (eGFR) was calculated using the Chronic Kidney Disease Epidemiology Collaboration (CKD-EPI) equation (Levey et al., 2009). Body mass index (BMI) was calculated as weight (kg)/height (meters)^2^. 

### Genotyping

Total DNA was extracted from peripheral blood samples using a standardized technique. *TGFB1* rs1800469 (c.-1347 C>T; C-509T) and rs1800470 (c.+29 T>C; T869C; Leu10Pro) SNPs were genotyped using TaqMan SNP Genotyping Assays 20X (Thermo Fisher Scientific, Foster City, CA, USA; Assay ID: C_8708473_10 and C_22272997_10, respectively). Real-Time PCR reactions were performed in 384-well plates, in a total 5 µL volume, using 2 ng of DNA, TaqMan Genotyping Master Mix 1X (Thermo Fisher Scientific) and TaqMan Genotyping Assay 1X. PCR reactions were performed in a real-time PCR thermal cycler (ViiA7 Real-Time PCR System; Thermo Fisher Scientific). 

### Haplotype distributions and linkage disequilibrium (LD) analysis

The haplotypes constructed by the combination of the rs1800469 and rs1800470 *TGFB1* SNPs and their frequencies were inferred using the Phase 2.1 program (Seattle, WA, USA), which implements a Bayesian statistical method (Stephens et al., 2001). We also used this program to compare the distributions of different *TGFB1* haplotypes between DR patients and control subjects through permutation analyses of 10, 000 random replicates (Stephens *et al.,* 2001). Linkage disequilibrium (LD) between the two SNPs was calculated using Lewontin´s *D´|D´| and r* ^*2*^ measurements (Hedrick 1987). 

### Statistical analyses

Allele frequencies were determined by gene counting, and departures from the Hardy-Weinberg Equilibrium (HWE) were assessed using the *χ* ^2^ test. Allele and genotype frequencies were compared between groups of subjects using *χ* ^2^ tests. Moreover, genotypes were compared between case and control groups considering additive, recessive, and dominant inheritance models (Zintzaras and Lau, 2008). Normal distributions of quantitative clinical and laboratory variables were checked using Kolmogorov-Smirnov and Shapiro-Wilk tests. Variables with normal distribution are shown as mean ± SD. Variables with skewed distribution were log-transformed before analysis and are shown as median (25th - 75th percentile values). Categorical data are shown as percentages.

Clinical and laboratory characteristics were compared between case and control patients and between groups of patients categorized according to the different genotypes of the two *TGFB1* SNPs using appropriate statistical tests, such as Student’s *t-*test or *χ* ^2^ tests. Bonferroni’s correction was applied to account for multiple comparisons for unpaired Student’s *t* tests or *χ* ^2^ tests.

The magnitude of association between *TGFB1* SNPs and DR was estimated using odds ratios (OR) with 95% confidence intervals (CI). Multivariate logistic regression analyses were done to evaluate the independent association of each individual *TGFB1* SNP or haplotypes with DR, adjusting for possible confounding factors. Statistical analyses were performed using the SPSS 18.0 software (SPSS, Chicago, IL), and P values < 0.05 were considered significant. Sample size was calculated using the OpenEpi site (http://www.openepi.com) and the minor allele frequencies and ORs observed in previous studies regarding associations of the rs1800469 and rs1800470 SNPs with DR (Beránek et al., 2002; Paine et al., 2012; Rodrigues et al., 2015).

## Results

### Sample description

The clinical and laboratorial characteristics of DR cases and controls are shown in Table 1. Males comprised 52.6% of the case group and 44.2% of the control group (P = 0.010), and the mean age was 62.5 ± 15.1 years in cases and 59.5 ± 20.1 in controls (P = 0.010). The mean DM duration was higher in cases compared to controls (23.3 ± 9.2 *vs*. 21.4 ± 9.0; P = 0.002). As expected, mean levels of LDL, triglycerides and UAE, as well as prevalence of AH were significantly higher in cases compared to control subjects (all P < 0.003). Ethnic distribution, BMI, HbA1c, total cholesterol, and HDL cholesterol levels did not differ significantly between groups (Table 1).

**Table 1 -:** Clinical and laboratory characteristics of DM patients without and with DR.

Characteristics	Controls (n = 446)	Cases with DR (n = 546)	P *
Age (years)	59.5 ± 20.1	62.5 ± 15.1	0.010
Gender (% males)	197 (44.2)	287 (52.6)	0.010
Ethnicity (% non-white)	64 (14.3)	91 (16.7)	0.358
T2DM patients (%)	305 (68.5)	422 (77.3)	0.002
DM duration (years)	21.4 ± 9.0	23.3 ± 9.2	0.002
BMI (kg/m²)	27.8 ± 5.2	27.9 ± 5.1	0.747
HbA1c (%)	7.8 ± 1.9	8.2 ± 2.1	0.015
Cholesterol total (mg/dL)	189.1 ± 49.0	198.0 ± 51.6	0.007
HDL cholesterol (mg/dL)	49.7 ± 14.0	48.5 ± 14.7	0.182
LDL cholesterol (mg/dL)	108.9 ± 41.4	117.6 ± 45.0	0.002
Triglycerides (mg/dL)	127.0 (75.0 - 189.0)	133.5 (32.7 - 86.0)	0.001
Arterial hypertension (%)	322 (72.2)	477 (87.4)	0.0001
eGFR (ml/min per 1.73 m^2^)	83.5 (61.0 - 100.0)	62.0 (32.7 - 86.0)	0.102
UAE (mg/g)	8.0 (4.0 - 30.5)	54.9 (9.3 - 381.5)	0.0001

Variables are shown as mean ± SD, median (25th-75th percentiles) or absolute number (%). *P-*values* were computed using Student’s *t* or *χ* ^2^ tests, as appropriate. Only*P*values lower than the Bonferroni’s threshold (*P*= 0.0035) were considered statistically significant. BMI: body mass index; DM: diabetes mellitus; DR: diabetic retinopathy; eGFR: estimated glomerular filtration rate; HbA1c: glycated hemoglobin; T2DM: type 2 diabetes mellitus; UAE: urinary albumin excretion.

### Distributions of the TGFB1 rs1800469 and rs1800470 SNPs in case and control groups

Genotype frequencies of the rs1800469 (c.-1347 C>T) and rs1800470 (c.+29 T>C) SNPs in the *TGFB1* gene are in HWE in the case group (all P > 0.05). Frequencies of rs1800469 T/T and rs1800470 C/C genotypes did not differ significantly between white and non-white subjects (rs1800469 T/T: 15.0 *vs*. 17.2%, respectively; P = 0.354; rs1800470 C/C: 20.8 *vs*. 25.8%, P = 0.188). Moreover, frequencies of these genotypes did not differ between T1DM and T2DM patients (rs1800469 T/T: 15.3 *vs*. 15.2%, respectively; P = 0.893; rs1800470 C/C: 19.9 *vs*. 22.1%; P = 0.595). Hence, both white and non-white subjects, as well as patients with T1DM and T2DM, were analyzed together. 

Table 2 shows genotype and allele frequencies of the rs1800469 and rs1800470 SNPs in patients with DM (T1DM + T2DM) categorized into DR cases and non-DR controls. Frequency of the T/T genotype of the rs1800469 SNP was 18.3% in controls and 12.7% in cases with DR (P = 0.022). After adjustment for HbA1c, AH, UAE, and triglycerides, the T/T genotype remained associated with protection against DR in the recessive model (OR = 0.604; 95% CI 0.395 - 0.923; P = 0.020). Regarding the rs1800470 SNP, the frequency of the C/C genotype was 25.4% in controls and 18.0% in cases with DR (P = 0.015). In the recessive model, the rs1800470 T/T genotype was also found to be associated with protection against DR, independent of the variables described above (OR = 0.589; 95% CI 0.405 - 0.857; P = 0.006).

**Table 2 -:** Genotype and allele frequencies of *TGFB1* rs1800469 and rs1800470 SNPs in DM patients without and with DR.

rs1800469	Controls (n = 437)	Cases with DR (n = 529)	Unadjusted P*	Adjusted OR (95% IC) / P†
**Genotype**				
C/C	182 (41.6)	214 (40.5)	0.022	1
C/T	175 (40.0)	248 (46.8)		1.267 (0.910 - 1.765)/ 0.161
T/T	80 (18.4)	67 (12.7)		0.680 (0.431 - 1.073)/ 0.097
**Allele**				
C	0.62	0.64	0.337	
T	0.38	0.36		
**Recessive model**				
C/C + C/T	357 (81.7)	462 (87.3)	0.019	1
T/T	80 (18.3)	67 (12.7)		0.604 (0.395 - 0.923)/ 0.020
**Additive model**				
C/C	182 (69.5)	214 (76.2)	0.098	1
T/T	80 (30.5)	67 (23.8)		0.656 (0.410 - 1.051)/ 0.079
**Dominant model**				
C/C	182 (41.6)	214 (40.5)	0.757	1
C/T + T/T	255 (58.4)	315 (59.5)		1.075 (0.790 - 1.462)/ 0.645
rs1800470	Controls (n = 426)	Cases with DR (n = 512)	Unadjusted P*	Adjusted OR (95% IC) / P†
**Genotype**				
T/T	136 (31.9)	165 (32.2)	0.015	1
T/C	182 (42.7)	255 (49.8)		1.255 (0.882 - 1.786)/ 0.207
C/C	108 (25.4)	92 (18.0)		0.673 (0.439 - 1.031)/ 0.069
**Allele**				
T	0.53	0.57	0.105	
C	0.47	0.43		
**Recessive model**				
T/T + T/C	318 (74.6)	420 (82.0)	0.008	1
C/C	108 (25.4)	92 (18.0)		0.589 (0.405 - 0.857)/ 0.006
**Additive model**				
T/T	136 (55.7)	165 (64.2)	0.065	1
C/C	108 (44.3)	92 (35.8)		0.674 (0.437 - 1.039)/ 0.074
**Dominant model**				
T/T	136 (31.9)	165 (32.2)	0.977	1
T/C + C/C	290 (68.1)	347 (67.8)		1.026 (0.740 - 1.423)/ 0.876

Data are shown as number (%) or proportion. *P-values were calculated using *χ* ^2^ tests. Only*P*values lower than the Bonferroni’s threshold (*P*= 0.010) were considered statistically significant. † P-value and OR (95% CI) obtained using logistic regression analyses adjusting for HbA1c, AH, UAE and triglycerides levels.

### Haplotype distributions and LD

Frequencies of haplotypes produced by the combination of *TFGB1* rs1800469 and rs1800470 SNPs in cases and controls are listed in Table 3. Four haplotypes were inferred in both samples and their distributions were not significantly different between case and control groups (P = 0.564). It is noteworthy that the two SNPs of interest are in partial LD in our population (|D′| = 0.679 and r^2^ = 0.335). 

**Table 3 -:** Haplotypes of the *TGFB1* SNPs in DM patients without and with DR.

Haplotypes	Controls	Cases with DR	P *
TT	0.086	0.088	0.564
TC	0.522	0.548	
CT	0.379	0.355	
CC	0.013	0.009	

Data are presented as proportion. The first letter of the haplotypes refers to the rs1800470 SNP and the second to the rs1800469 SNP. *Permutation P-value was computed for comparisons of haplotype frequencies between groups.

**Figure 1 -:**
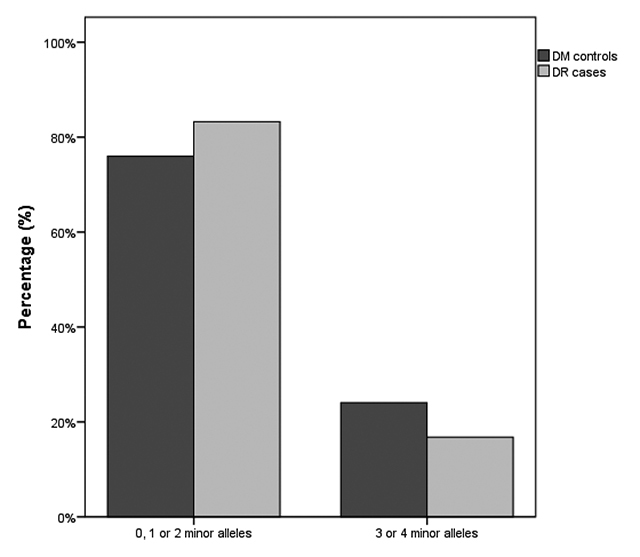
DR cases and DM controls were categorized by the number of risk alleles of the analyzed polymorphisms in the estimated haplotypes. Data are presented as percentage. P= 0.008 was obtained using the *χ* ^2^-test and considering the absolute number of patients in each category.

Next, in order to increase statistical power, we further analyzed haplotype frequencies according to the number of minor alleles in haplotypes: a) subjects carrying 0, 1 or 2 minor alleles of rs1800469 and rs1800470 SNPs, and b) subjects carrying 3 or 4 minor alleles (Figure 1). Frequency of 3 or 4 minor alleles of the two analyzed SNPs was lower in DR cases compared to controls (16.8% *vs*. 24.0; P = 0.008; Figure 1). Moreover, after adjustment for AH, HbA1c, UAE, and triglycerides levels, the presence of ≥3 minor alleles remained independently associated with protection against DR (OR = 0.549; 95% CI 0.371 - 0.812; P = 0.003). The observed OR is similar to those obtained for each SNP analyzed individually, suggesting that their effects on DR susceptibility may not be additive.

## Discussion

TGFB1 has been recognized as a key factor in the pathogenesis of chronic microvascular complications of DM (Jia et al., 2011; Liu et al., 2014). Accordingly, SNPs in the *TGFB1* gene have been shown to be involved in the susceptibility for DKD due to the role of this gene on tissue fibrosis processes (Buraczynska et al., 2007; Jia *et al.,* 2011; Zhou et al., 2018; Varghese and Kumar, 2019; Zhou *et al.,* 2019). Moreover, *TGFB1* SNPs seem to be associated with susceptibility for DR (Paine et al., 2012; Liu *et al.,* 2014; Hampton et al., 2015); however, available data is less convincing. Thus, in this study, we investigated the association of *TGFB1* rs1800469 and rs1800470 SNPs with DR in T1DM and T2DM patients from a Southern Brazilian population. Our findings suggest that both SNPs are associated with protection against DR. 

The rs1800469 SNP (c.-1347 C>T; also known as C-509T) is situated in the first negative regulatory region of the upstream promoter of the *TGFB1* gene, and the T allele seems to increase both *TGFB1* gene expression and circulating plasma levels in humans (Grainger et al., 1999; Shah et al., 2006; Martelossi Cebinelli et al., 2016). Interestingly, TGFB1 concentration seems to be higher in T/T homozygous than heterozygous, suggesting a dose-response effect (Grainger *et al.,* 1999). Elevated TGFB1 plasma levels have been associated with the progression of renal disease due to increased ECM production, leading to glomerulosclerosis and tubulointerstitial fibrosis (Loeffler and Wolf, 2014). In the context of DR pathogenesis, augmented TGFB1 circulating levels might enhance angiogenesis and endothelial proliferation, as well as ECM production and blood-retina barrier breakdown, thereby contributing to the development and progression of DR (Khan and Chakrabarti, 2003; Jia et al., 2011; Liu et al., 2014).

Besides functional studies reporting the impact of the rs1800469 T allele on TGFB1 levels, the association of this SNP with diabetic chronic complications remains inconclusive. Our present case-control study demonstrated a significant association of the T/T genotype with protection against DR. Consistent with our findings, the C allele of this SNP was found to be more prevalent in PDR patients (P = 0.050), and this allele was associated with risk of PDR in the haplotype constituted together with the rs1800470 SNP (Beránek et al., 2002). In contrast, the meta-analysis conducted by Liu et al. (Liu et al., 2014) did not reveal any significant association between this SNP and DR. These discrepant findings may be explained by differences in ethnicities since the studies included in the meta-analysis involved T2DM patients from Czech, Poland, and India populations (Liu *et al.,* 2014). Moreover, the meta-analysis only included 3 studies comprising 521 T2DM patients with DR and 580 controls, raising the possibility of insufficient statistical power. Furthermore, Raina et al. (2015) demonstrated that the T/T genotype of rs1800469 SNP was associated with a 5.5-fold increased risk of end-stage renal disease (ESRD) in T2DM patients from North India. However, other studies have not been able to find any association between this SNP and DKD (Ng et al., 2003; McKnight et al., 2007; Prasad et al., 2007). Although functional studies suggest that the rs1800469 T allele leads to worse outcomes related to the pathogenesis of microvascular diabetic complications, the results of case-control studies that investigated this SNP in DM patients are still contradictory. Therefore, more studies with larger sample sizes are necessary to better understand the involvement of the rs1800469 SNP in DM and DR susceptibility. 

The rs1800470 SNP (c.+29 T>C; also known as T869C) causes the replacement of a Leucine (Leu) to a Proline (Pro) in codon 10 (Leu10Pro) of exon 1, which encodes the N-terminal signal peptide of TGFB1 (Martelossi Cebinelli et al., 2016). Although it has been speculated that modifications in amino acid composition of the signal peptide can affect its polarity and lead to different rates of protein export (Wood et al., 2000), both T and C alleles encode nonpolar amino acids (Martelossi Cebinelli *et al.,* 2016), suggesting they have similar effects on protein function. An *in vitro* study showed that the C (Pro) allele caused an increase in TGFB1 secretion compared to the T (Leu) allele (Dunning et al., 2003). Moreover, studies have shown that serum TGFB1 concentration is higher in subjects with the C/C genotype compared to T allele carriers (Yokota et al., 2000; Taubenschuss et al., 2013; Martelossi Cebinelli *et al.,* 2016). However, Ramirez et al. (2020) demonstrated that individuals carrying the T/T genotype have higher levels of TGFB1 when compared to C/C carriers. Hence, although the functional effect of this SNP on *TGFB1* expression is not yet clear, higher levels of TGFB1 can increase angiogenesis, ECM production, and blood-retina breakdown, thus predisposing to DR.

Our present study reported an association between the C/C genotype of the rs1800470 SNP and protection against DR. Supporting a protective role of the C allele, Javor et al. (2010) demonstrated an association between the T/T genotype and an increased risk for DR in T1DM patients from a Slovak population. Similarly, another study showed that the T allele is associated with risk for PDR (OR = 2.89; 95% CI 1.6 - 5.1) in T2DM patients from the Czech Republic (Beránek et al., 2002). In contrast, Buraczynska et al., (2007) reported that the C allele of this SNP was associated with increased risk of DR (OR = 2.22; 95% CI 1.64 - 2.99) in T2DM patients from Poland. Bazzaz et al. (2014) also reported that the frequency of the C allele was higher in T1DM patients with DR compared to controls, although the difference did not reach statistical significance. Moreover, a small study of Brazilian T2DM patients (66 cases with DR and 36 controls) did not find any significant association between the rs1800470 SNP and DR (Rodrigues et al., 2015). 

In 2011, Jia et al. (2011) published a meta-analysis of nine studies (1776 cases and 1740 controls) investigating the association between the rs1800470 SNP and DKD in T1DM or T2DM patients, which suggested that the presence of the C allele was associated with an increased risk for DKD (OR = 1.25, 95% CI 1.05 - 1.48). A recent meta-analysis of eight Chinese studies (1018 cases with DKD and 941 controls) reported that the T/T genotype conferred protection against DKD (OR = 0.55, 95% CI 0.31 - 0.96) in T2DM patients (Zhou et al., 2018). Despite the new data generated by our article, the results remain contradictory, and additional studies are necessary to clarify the association between this SNP and DR. 

This study has a few limitations. First, even though ethnic distributions were similar between case and control groups, there is a possibility of population stratification bias when analyzing the samples. Second, although the frequencies of the rs1800469 and rs1800470 SNPs were similar between T1DM and T2DM patients, the sample size was not sufficient to conduct further stratification analysis by DM type. Therefore, the possibility that the strength of association of these SNPs with DR might be different between DM types cannot be ruled out. Third, due to small sample sizes and the number of independent variables included in the models, corrections for multiple comparisons were not applied in logistic regression analyses. Thus, further studies are needed to confirm the results of this study.

In conclusion, this study suggests that the *TGFB1* rs1800469 and rs1800470 SNPs may confer protection against DR in T1DM and T2DM patients from Southern Brazil. Nevertheless, further research is necessary to confirm the role of these SNPs in the development of DR.
